# Supplementary material for: Evaluating intra- and inter-individual variation in the human placental transcriptome
Source: Genome Biol. 2015 Mar 19;16(1):54. doi: 10.1186/s13059-015-0627-z (PMC4404591; doi:10.1186/s13059-015-0627-z)
Supplement: Additional file 1: — Table and Figures: contains all supplementary figures and tables. [file 13059_2015_627_MOESM1_ESM.docx]

***Supplementary Figure and Tables***

**The Influence of Selection and Biological Traits on Human Placental Transcriptome Variation**

David A Hughes, Martin Kircher, Zhisong He, Guo Song, Genevieve L. Fairbrother, Carlos S. Moreno, Philipp Khaitovich, Mark Stoneking

# Figure S1: Read Counts


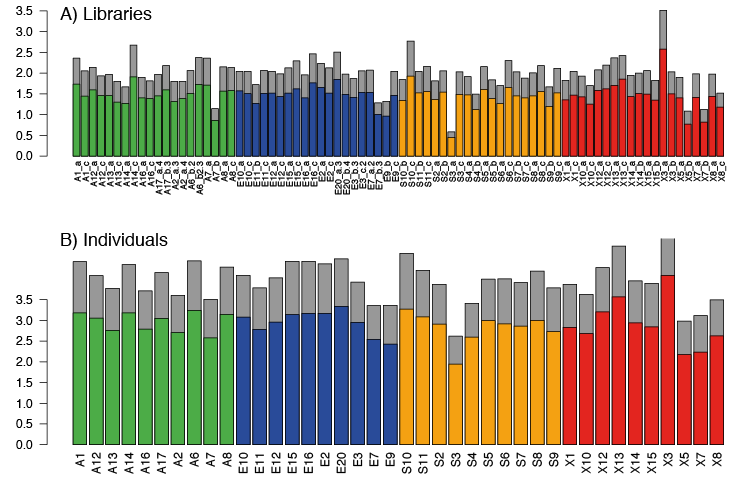


Read count bar plots for each library (A) and each individual (B). The total bar height represents the total number of quality RNA-Seq reads (in millions of reads) and the colored area represents the total number of mapped reads for each library and individual. Libraries and individuals are colored by population association: Green : African, Blue : European, Orange : South Asian, Red : East Asian.

# Figure S2: Validation Violin Plots

Violin plots of the original RNA-Seq data and rt-qPCR data for three DE genes. Real time quantitative PCR was carried out using sample replicates from the same individuals as the original study but from (1) a second aliquot from one of the originally used RNAs and (2) an RNA aliquot from a third sampling of the same placentas and thus an RNA isolate not used in the original study. Violin plots were generated from data at the library level. Pearson’s correlations and p-values are along the right side of plot; calculated using the mean value across replicates for each individual.

# Figure S3: Dot plots of the correlation among apportionment parameters.


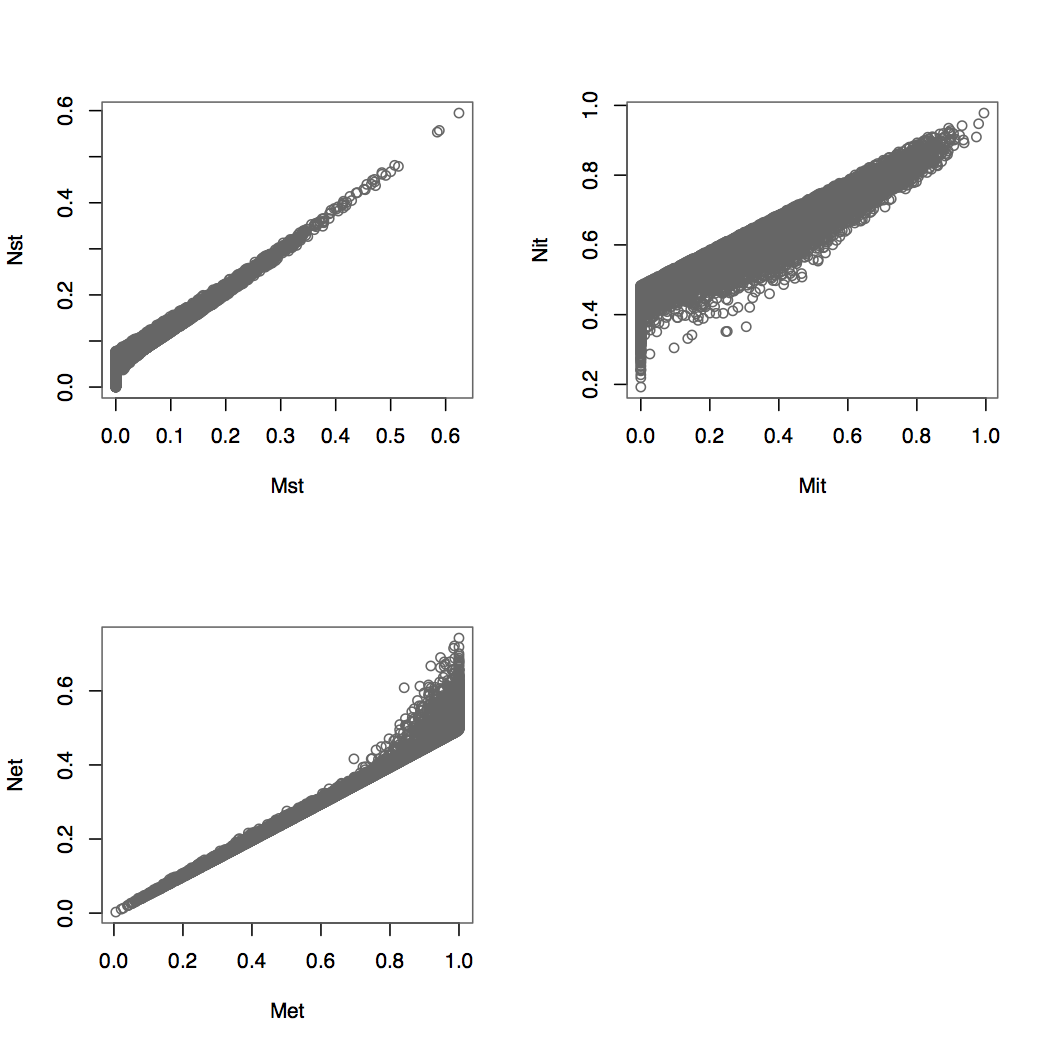


Correlation plots of the following parameters related to the apportionment of variance: among group (Mst:Nst); among individual (Mit, Nit); and within individual (Met:Net).

# Figure S4: Principle Component analysis of DE genes.


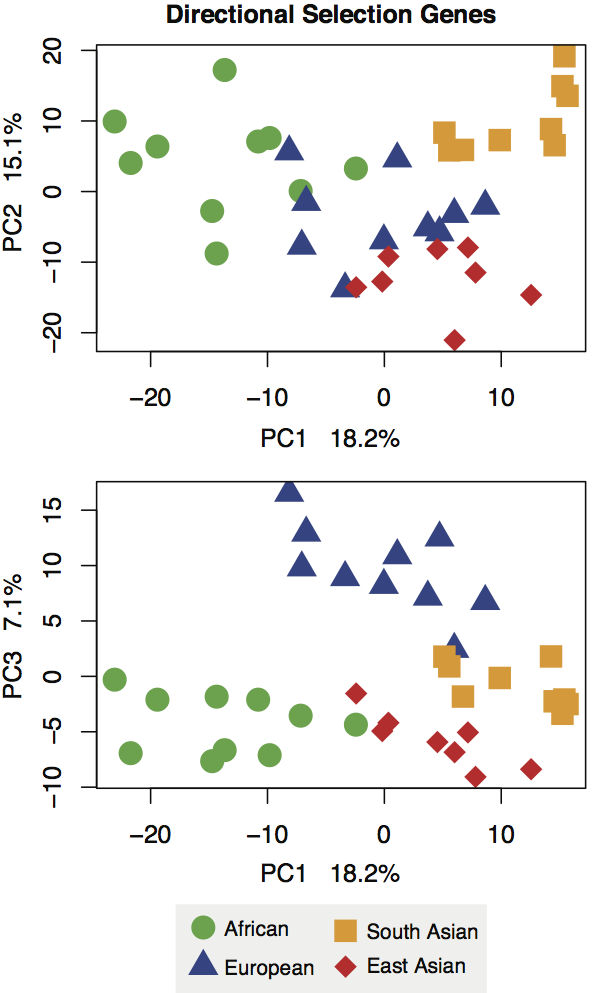


Scatter plot of principle components derived from an analysis of DE genes. All populations were modeled simultaneously in a single model, identifying 646 genes. PCs 1-3 are illustrated along with the proportion of variation explained by each component.

# Figure S5: Expression Variance and Genetic Diversity


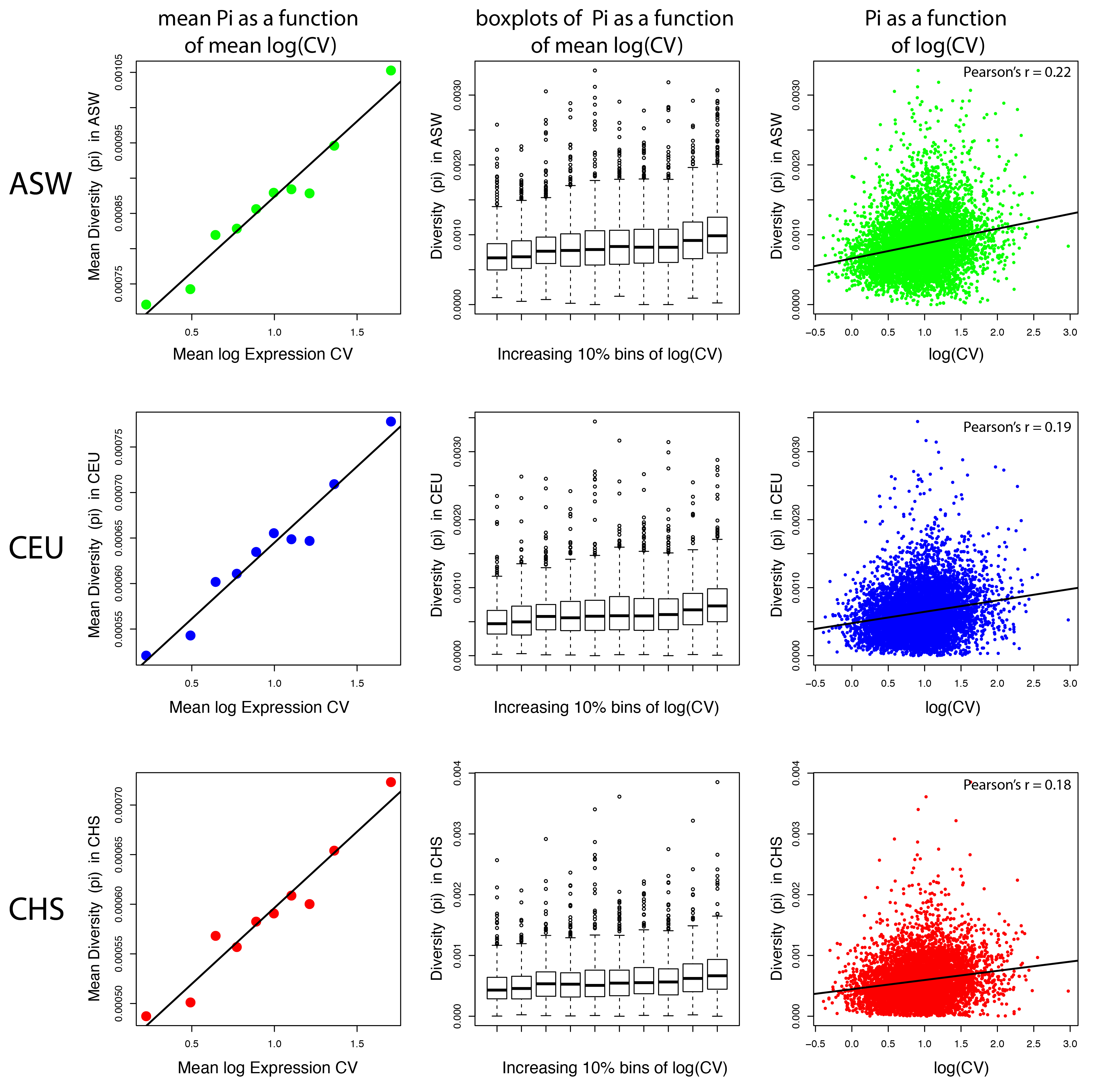


Plots illustrating the relationship between genetic diversity at a gene and its expression variance, or specifically the coefficient of variance (CV) in gene expression. Data are presented for three 1000genomes populations (African Americans (ASW), Europeans (CEU) and Southern Han Chinese (CHS)) presented on each row of the figure. Each column of the figure is an alternative representation of the same data. Column one illustrates how mean diversity (pi) increases with increasing bins (10 percentile intervals) of the log CV. Column two presents box plots of the diversity distribution within each bin. Colum three presents all the data as a scatter plot. It is clear that the effect is small, with an r-squared of roughly 0.04, but it remains a significant positive effect.

# Figure S6: Expression Variance and Degree of Connectivity


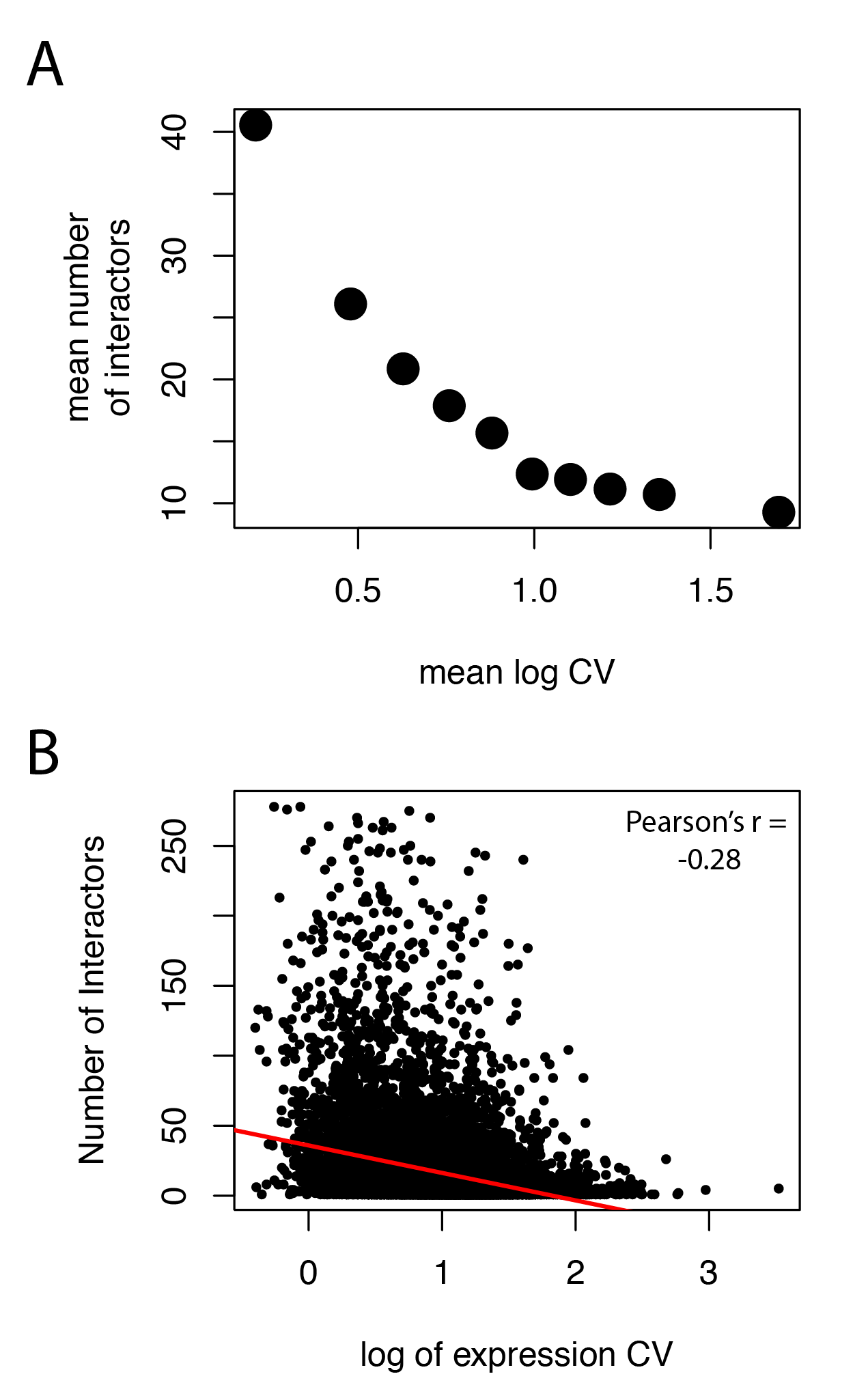


# Correlation plot between the mean of the log coefficient of variation in gene expression and the mean number of interacting genes. To generate this plot we sampled increasing bins (10 percentile intervals) of log CV and then for that set of genes averaged the number of interacting genes this distribution of genes had. (B) A density plot of all the data used to generate plot (A). The red line is a fitted linear regression. Figure S7: Kmeans results for directional or DE genes


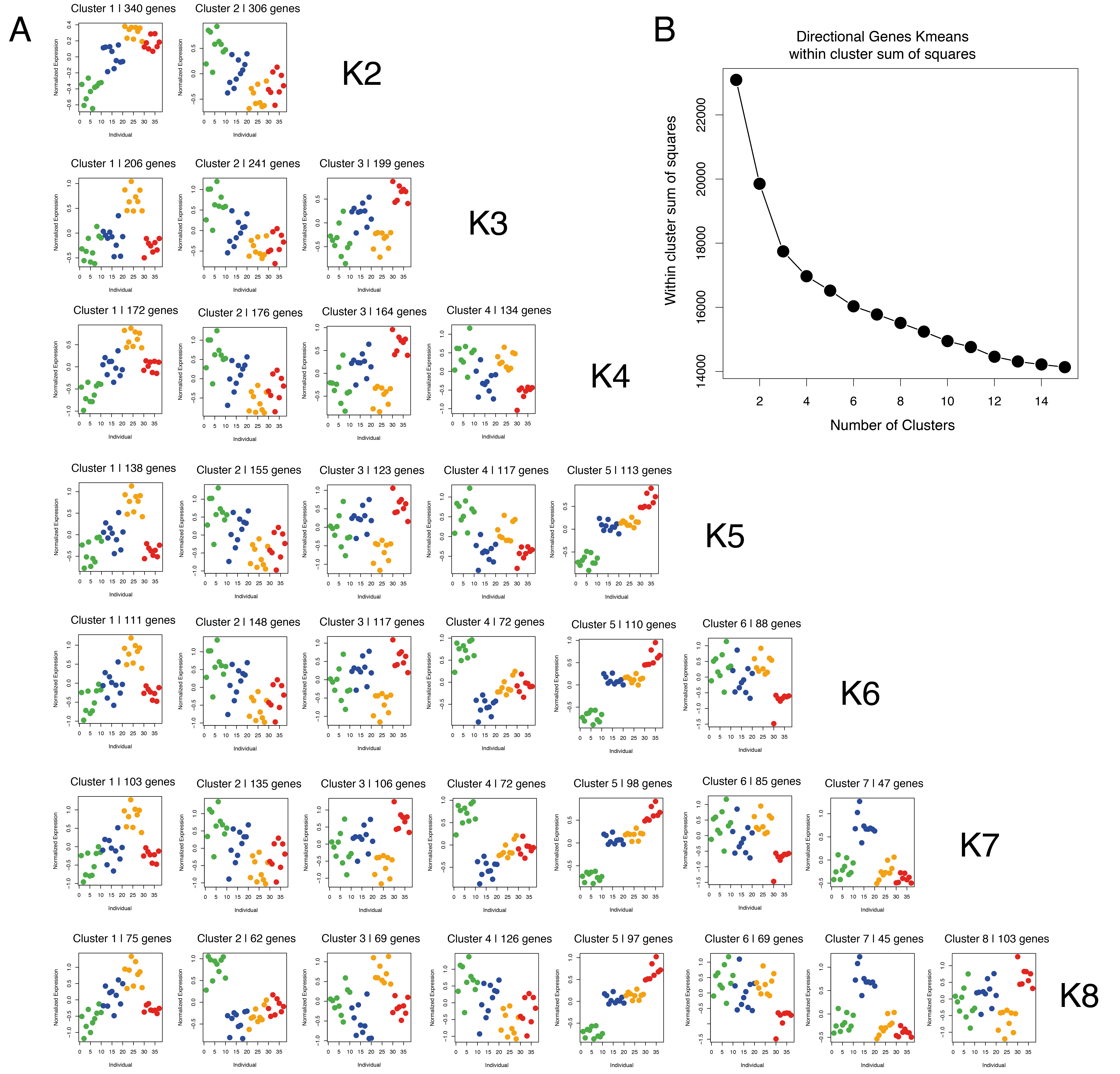


(A) Results for the Kmeans clustering algorithm as implemented in the stats package in R, where K (the number of clusters) was set from 2 to 8. The x-axis of the data demarcates each individual, and the y-axis is normalized gene expression. Genes were normalized to have a mean 0 and standard deviation of 1. Green, blue, orange and red colors denote associated ancestry to Africa, Europe, South Asia and East Asia, respectively. (B) The kmean algorithm attempts to limit the within group sums of squares, thereby maximizing the variance among clusters. This plot, illustrates the proportion of variance explained within clusters, as the number of clusters increase. Similar to a PCA Scree plot one might look for the “elbow” in the plot to identify the most informative number of clusters. Using those criteria, K3 may be the most informative K. Figure S8: Mean expression and Inter-individual effects on explanatory trait significance


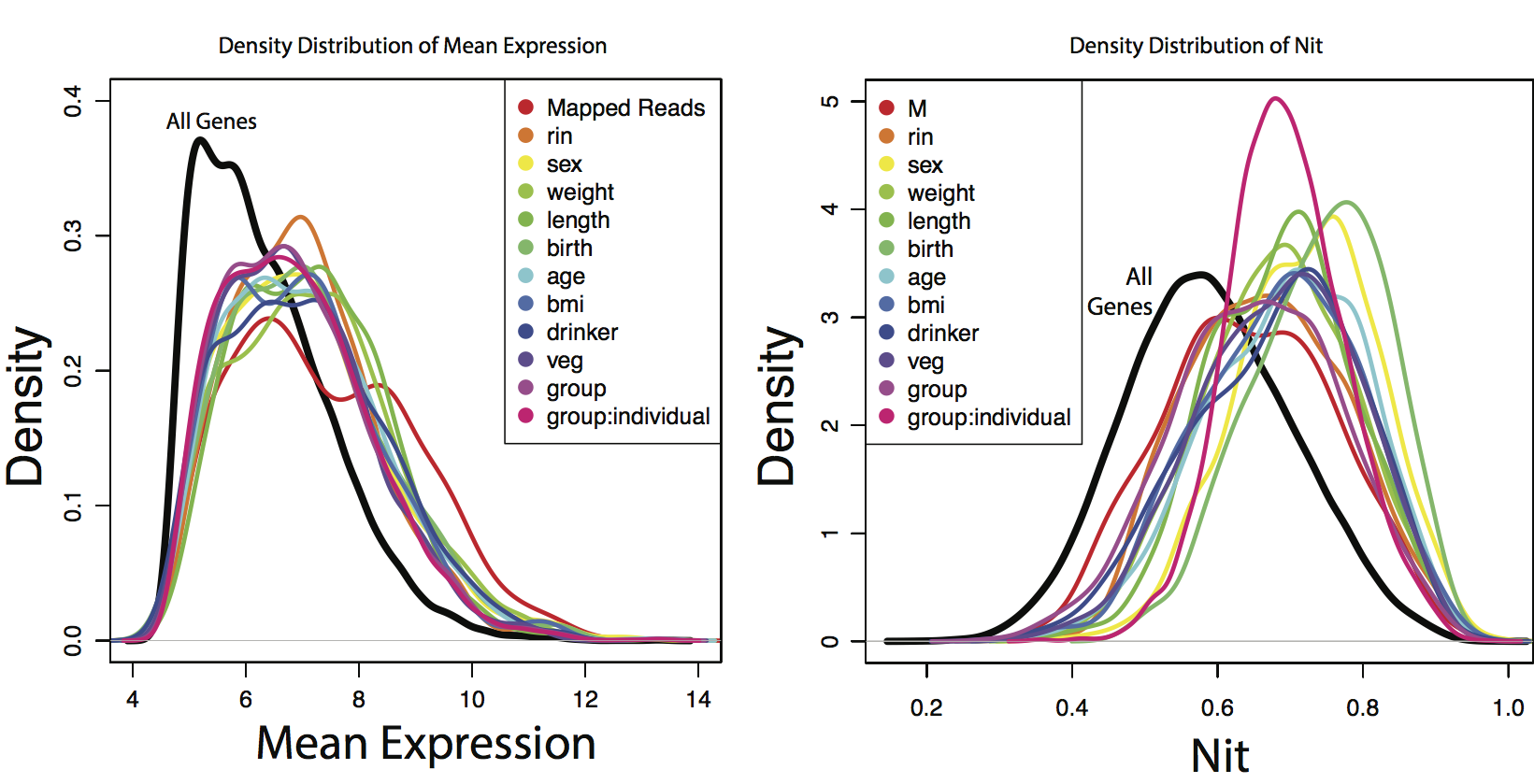


Density plots of mean expression and Nit for all genes and those genes significant by F-test for explanatory variables in the full model of gene expression. Shifts in the distributions, both for mean expression and Nit (inter-individual variation), influence the power at which genes were determined to be significant for each explanatory variable. In the mean expression plot (left), the technical variable (the number of Mapped Reads) was particularly influenced by mean expression.

| **Paramater** | **Formula** | **Definition** |
| --- | --- | --- |
| **Mst** | σ^2^/σ^2^_T_ | Proportion of total variance explained by groups |
| **Mit** | σ2/σ^2^_T_ | Proportion of total variance explain by individuals |
| **Met** | σ^2^/σ^2^_T_ | Proportion of total variance explain by sample replicates and error |
|  |  |  |
| **Net** | SS_A_/SS_T_ | Proportion of total variation explained by groups |
| **Nit** | SS**_B_**/SS_T_ | Proportion of total variation explain by individuals |
| **Net** | SS_e_/SS_T_ | Proportion of total variation explain by sample replicates and error |
| **Nis** | SS_B_/ (SS_B_ + SS_e_) | Proportion of variation found among sample replicates and among individuals explained by variation among individuals |
| **Nig** | SS_B_/ (SS_A_ + SS_B_) | Proportion of variation found among individuals and among groups explained by variation among individuals |

# **Table S1: Summary of Each Apportionment Parameter**

A quick guide table for each variation and variance parameter. We provide the formula for each parameter and its definition as we used it in the main text. See table S2 for clarity on the formulas.

# Table S2: Nested ANOVA TABLE

| Factor | Factor Abreviation | Source of Variation | SS | df | MS | EMS | Variance Component | Additive Variance Estimate |
| --- | --- | --- | --- | --- | --- | --- | --- | --- |
| group | A | among groups | SS_A_ | a-1 | MS_A_ | σ^2^ + n_0_σ^2^_B_ + nbσ^2^_A_ | σ^2^_A_ | σ^2^_A_ = (MS_A_ - MS_B_)/n_0_b |
| individual | B | among individuals within group | SS_B_ | a(b-1) | MS_B_ | σ^2^ + n_0_σ^2^_B_ | σ^2^_B_ | σ^2^_B_ = (MS_B_ - MS_e_) /n_0_ |
| sample replicate | E | among sample replicates (within individuals) | SS_E_ | ab(n_0_-1) | MS_e_ | σ^2^ | σ^2^ | σ^2^ = σ^2^ |
| Total | T | total variation | SS_T_ = SS_A_ + SS_B_ + SS_E_ | abn - 1 |  |  | σ^2^_T_ | σ^2^_T_ = σ^2^_A_ + σ^2^_B_ + σ^2^ |

An ANOVA table outlining our linear model for expression variation, the components of variation (SS) and variance (σ^2^) and formulas used to estimate the additive component of variances.

# Table S3: Primers for rt-qPCR Validation

| Primer name | Sequence | Product size |
| --- | --- | --- |
| CD97_F | CTTGGTGCTGACCTATGTG | 100bp |
| CD97_R | GTATTCTTCCCGAACCTTCT | 100bp |
| C19orf28_F | TGAGCTTCTTGGATAAGGTG | 110bp |
| C19orf28_R | CCAGTGGTAAAAGCTCACG | 110bp |
| UCA1_F | ATAAAAGTAGCGGCACAAAG | 90bp |
| UCA1_R | TTTTCTGTCACCTTTTCACA | 90bp |

Primer names, sequences and product sizes for rt/qPCR validation
